# Varying outcomes of triple-negative breast cancer in different age groups–prognostic value of clinical features and proliferation

**DOI:** 10.1007/s10549-022-06767-1

**Published:** 2022-10-19

**Authors:** H. Vihervuori, K. Korpinen, T. A. Autere, H. Repo, K. Talvinen, P. Kronqvist

**Affiliations:** 1 Institute of Biomedicine, University of Turku, Kiinamyllynkatu 10, 20510 Turku, Finland; 2 Department of Pathology, Turku University Hospital, Turku, Finland; 3 Department of Pathology, Central Hospital of Central Finland, Jyväskylä, Finland

**Keywords:** Triple-negative breast cancer, Prognosis, Survival, Age, Proliferation, Ki-67, Geminin

## Abstract

**Purpose:**

Triple-negative breast cancer (TNBC) is an aggressive disease lacking specific biomarkers to guide treatment decisions. We evaluated the combined prognostic impact of clinical features and novel biomarkers of cell cycle-progression in age-dependent subgroups of TNBC patients.

**Methods:**

One hundred forty seven TNBC patients with complete clinical data and up to 18 year follow-up were collected from Turku University Hospital, Finland. Eight biomarkers for cell division were immunohistochemically detected to evaluate their clinical applicability in relation to patient and tumor characteristics.

**Results:**

Age at diagnosis was the decisive factor predicting disease-specific mortality in TNBC (*p* = 0.002). The established prognostic features, nodal status and Ki-67, predicted survival only when combined with age. The outcome and prognostic features differed significantly between age groups, middle-aged patients showing the most favorable outcome. Among young patients, only lack of basal differentiation predicted disease outcome, indicating 4.5-fold mortality risk (*p* = 0.03). Among patients aged > 57, the established prognostic features predicted disease outcome with up to 3.0-fold mortality risk for tumor size ≥ 2 cm (*p* = 0.001). Concerning cell proliferation, Ki-67 alone was a significant prognosticator among patients aged > 57 years (*p* = 0.009). Among the studied cell cycle-specific biomarkers, only geminin predicted disease outcome, indicating up to 6.2-fold increased risk of mortality for tumor size < 2 cm (*p* = 0.03).

**Conclusion:**

Traditional clinical features do not provide optimal prognostic characterization for all TNBC patients. Young age should be considered as an additional adverse prognostic feature in therapeutic considerations. Increased proliferation, as evaluated using Ki-67 or geminin immunohistochemistry, showed potential in detecting survival differences in subgroups of TNBC.

## Introduction

Breast cancer presents as distinctive subtypes with various behavioral patterns. Among these, triple-negative breast cancer (TNBC) remains a major challenge since it affects younger patients than the other subtypes and inflicts a particularly aggressive course of disease. Consequently, the average 5 year survival rate is reported to be considerably lower among TNBC patients (77%) than among breast cancer patients in general (91%) (American Cancer Society 2022).

TNBC comprises several tumor subtypes with varying biological characteristic and behavioral patterns [1]. Recently, targeted therapies, i.e. immune checkpoint, AKT pathway and PARP inhibitors, have provided promising treatment options for subgroups of TNBC patients with high mutational burden and germline BRCA mutations [2]. Still, in the majority of TNBC cases, no clinically applicable expression signatures are available for identifying the prognosis of individual patients [3]. In clinical practice, international guidelines for treatment decisions of TNBC are based on the traditional prognostic features of breast cancer, i.e. stage, patient’s age at diagnosis and, to some extent, the proliferative activity of the malignant cells [4]. In most cases, the course of disease of a single TNBC patient is still unpredictable and, therefore, there is a constant need for intensified, clinically applicable, personalized prognostic markers.

Uncontrollable proliferation is one of the crucial properties of malignant transformation [5]. Mitotic activity is an established prognostic feature reflecting tumor biology also in breast cancer, especially in the luminal subtype [1, 6]. Ki-67 is the routinely applied proliferation marker but only few studies have addressed Ki-67 as a prognostic feature in TNBC [7–9], part of them showing controversial results [10]. The clinical applicability of Ki-67 in predicting the outcome of TNBC is limited due to the lack of standardized cut-off values [7, 11]. In previous literature, the applied optimal cut-points for Ki-67 labeling index have ranged from 10 to 61% [11–13]. Moreover, quantification of Ki-67-immunoexpression has been criticized for poor inter- and intra-observer concordance [11, 14].

Ki-67 does not only identify cells actually progressing into mitosis. Instead, it is expressed with varying functions throughout the cell cycle from S to G0 phases [15]. Previous literature presents several attempts to intensify the evaluation of cell proliferation by detecting proteins with specific functions and precise expression profiles in the cell cycle [16]. Among them, the metaphase-anaphase transition is one of the points-of-no-return in the cell cycle, where sister chromatids, after perfect alignment at the equator of the cell, separate and move to opposite poles of the mitotic spindle to start final division of the genetic material [17]. Several checkpoints participate in quality control at this event by monitoring and maintaining the accuracy of the DNA replication and repair, proper bi-orientation, and separation of the chromatids. Any disturbed regulatory cascades will lead to uncontrolled cell division and potentially induce excessive cell proliferation and malignant progression. Disrupted control mechanisms may result in mis-segregation of chromosomes, predisposing the cells to the loss of tumor-suppressor genes, chromosomal instability, and carcinogenesis [18].

In this paper, we evaluate the prognostic potential of clinical features and cancer cell proliferation among TNBC patients. For this purpose, we selected nine proliferation markers to investigate their prognostic potential and clinical applicability. These markers include the established mitotic indices Ki-67 and MCM2, as well as a set of regulatory proteins which have previously shown prognostic potential in assessing the outcome of breast cancer, i.e. geminin, separase, PLK1, aurora A, securin, cyclinB1 and CDK1-3. The results emphasize the biological and clinical heterogeneity of TNBC, and the significance of multifactorial prognostic evaluations in this breast cancer subgroup.

## Material and methods

### Patient and tissue material

In total, the study comprises 147 TNBC patients diagnosed and treated between 2000 and 2015 in Turku University Hospital, Turku, Finland (Table 1). The cases were identified based on surrogate markers of molecular subclassification according to WHO tumor classification criteria and St. Gallen consensus [1, 19]. All patients were treated with either conservative breast surgery or mastectomy combined with either sentinel node investigation or axillary evacuation. Surgery was followed by radiation and/or cytostatic treatment based on the international guidelines of TNBC at the time of diagnosis [20]. Patients detected with distant metastases at the time of diagnosis were excluded from the material. None of the patients received neoadjuvant treatment. Clinical information and complete follow-up data of the patients was provided by Auria Biobank (Auria Biobank, Turku University Hospital, Turku, Finland, https://www.auria.fi/biobank/). Causes of death were obtained from autopsy reports, death certificates and Finnish Cancer Registry (Statistics Finland, Helsinki, Finland), resulting in maximum follow-up period of 18 years (mean 8 years).

**Table 1:** Clinical features of the patient material (*n* = 147)

Age at diagnosis (years), mean (range)	60 (27 − 92)
Tumor size (diameter, cm), mean (range)	2.5 (0.5 − 8.5)
Node positive (%)	33
Basal differentation (%)	83
Breast cancer mortality during follow-up period (%)	22.4
Survival (years), mean (range)	8 (0.1 − 18)

The tissue specimen were obtained from Auria Biobank. All tumors represented poorly differentiated invasive ductal breast cancer NST. Originally, the specimen were treated according to standard pathology procedure, fixed in formalin (pH 7.0) and embedded in paraffin. Two specialists in breast pathology reassessed the specimen and selected the representative areas from HE-stained whole tumor sections. Thereafter, the tissue material was arranged in tissue microarrays (TMAs) by punching two 1.5 mm diameter cylinders from each tumor; one from the central and another from the peripheral tumor area. The TMAs were constructed using an automated tissue arrayer (TMA Grand Master machine, 3D HISTOTECH, Budapest, Hungary).

### Immunohistochemical methods

Triple-negativity of the tumors was confirmed by the absence of receptors for estrogen (ER) and progesterone (PR) using standard IHC practice, and HER2 amplification based on IHC alone (scores 0 or 1 +) or the combination of IHC (score 2 +) with negative amplification status detected in double in situ hybridizations (HER2 and chromosome 17 probes) [1]. Basal differentiation of the tumor cells was detected based on immunoexpression of epidermal growth factor receptor (EGFR) and/or cytokeratins 5/6, according to international guidelines [1, 21].

Immunohistochemical detection of the studied proteins (Table 2) followed the standard, previously described procedures [22].

**Table 2:** Summary of applied immunohistochemical methods

	Clone	Source	Pretreatment	Dilution	Device
Aurora A	EP1008Y	Abcam	MW^a^ pH9	1:500	Labvision^d^
Geminin	EPR14637	Abcam	MW pH9	1:1000	Labvision
MCM2	BS18	BioSite	MW pH9	1:800	Labvision
PLK1	36–298	Invitrogen	MW pH9	1:4000	Labvision
Securin	DCS-280	Abcam	MW pH6	1:100	Labvision
Separase	6H6	Abnova	MW pH6	1:1000	Labvision
CDK1-3	E161	Abcam	HIER CC1^b^	1:500	Discovery^e^
cyclinB1	Y106	Abcam	HIER CC1	1:100	Discovery
Ki-67	30–9	Roche	HIER CC1	RTU^c^	Benchmark^f^

^a^MW = microwave oven

^b^HIER CC1 = heat-induced epitope retrieval, commercial reagent, performed on automated platform

^c^RTU = Ready-to-use

^d^Labvision Autostainer (Thermo Fisher Scientific, Fremont CA USA)

^e^Discovery XT (Roche Diagnostics/Ventana Medical Systems, Tucson, AZ, USA)

^f^Benchmark XT (Roche Diagnostics/Ventana Medical Systems, Tucson, AZ, USA)

**Fig. 1:**
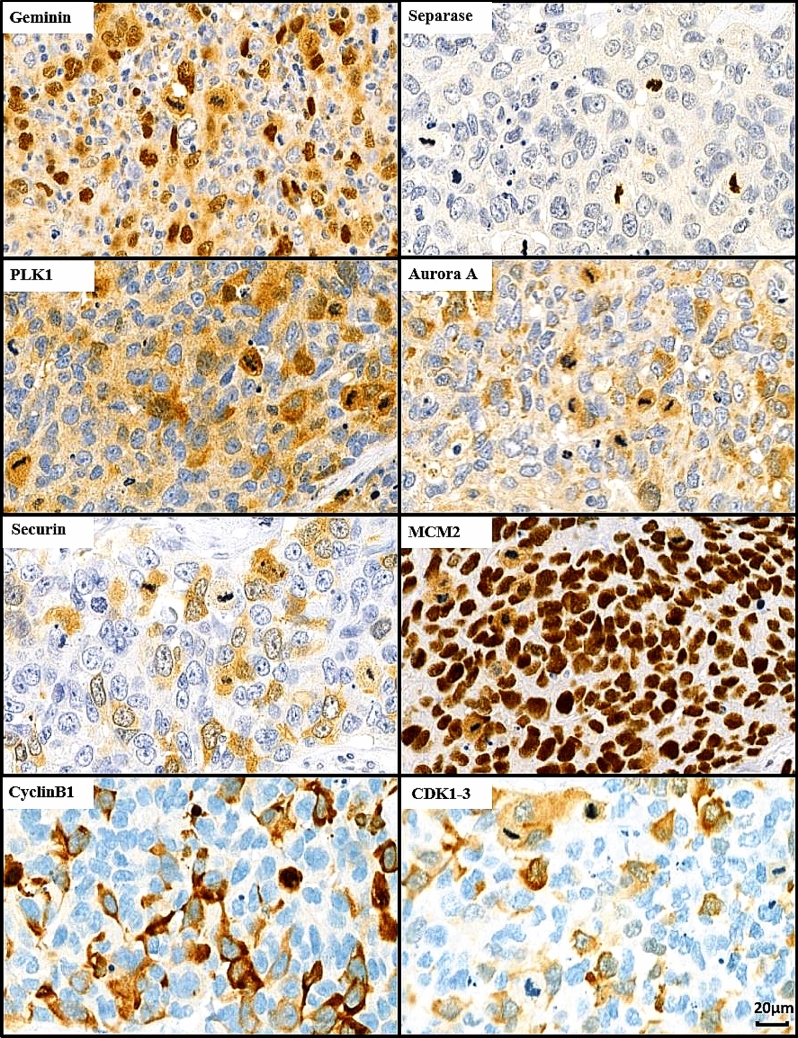
Summary of the immunoexpression patterns of the studied proliferation markers geminin, separase, PLK1, aurora A, securin, MCM2, cyclinB1 and CDK1-3 in consecutive sections of a single breast cancer specimen

The expression profiles of the studied proliferation markers are demonstrated in Fig. 1. In each tissue core of the TMAs, 1–3 foci consisting of at least 100 invasive cancer cells were selected for immunoevaluations. Cores with less than 100 invasive cancer cells or suboptimal tissue preservation were excluded from the analysis. The fraction of positively stained cancer cells was counted from each chosen field and the mean value was registered. In each patient case, the highest value of the two TMA cores, either the central or peripheral core, was applied in further analysis. The evaluations were performed using an automated image analysis software ImmunoRatio (https://biii.eu/immunoratio, Institute of Biomedical Technology, University of Tampere, Tampere, Finland) when applicable [23]. Immunopositivity was originally detected in each staining based on visual observation and, thereafter, the set criteria were applied to ImmunoRatio.

### Statistical analysis

In statistical analyses, the established clinical prognostic factors were assessed as continuous variables and classified according to internationally accepted criteria [1]. Patients’ age at diagnosis was statistically divided at median value (57 years) and at first and third quartiles (49 and 73 years, respectively). The studied proliferation markers were analyzed both as continuous and classified variables which categorized the patients into subgroups exhibiting low versus high expression of the studied proteins. This was performed by selecting cut-points based on, first, the observations of the immunohistochemical expression patterns and, secondly, on statistical analyses involving the mean, median and quartile values of each parameter. The optimal cut-point for each protein was verified based on univariate analyses identifying the cut-point producing the most significant survival difference between cancer-specific survival versus mortality in our material. Prognostic associations were modelled for the clinical prognostic features and the studied proliferation markers using Kaplan–Meier plots and Cox’s regression models detecting associations for breast cancer-specific mortality. Each association was quantitated as hazard ratios (HR) with 95% confidence intervals (CI). *p* values under 0.05 were considered significant. Statistical analysis was performed using R Statistical Software (R Development Core Team, 2017) and Cox regression models with the 'survival' package [24] and 'Survminer' package [25].

## Results

**Fig. 2:**
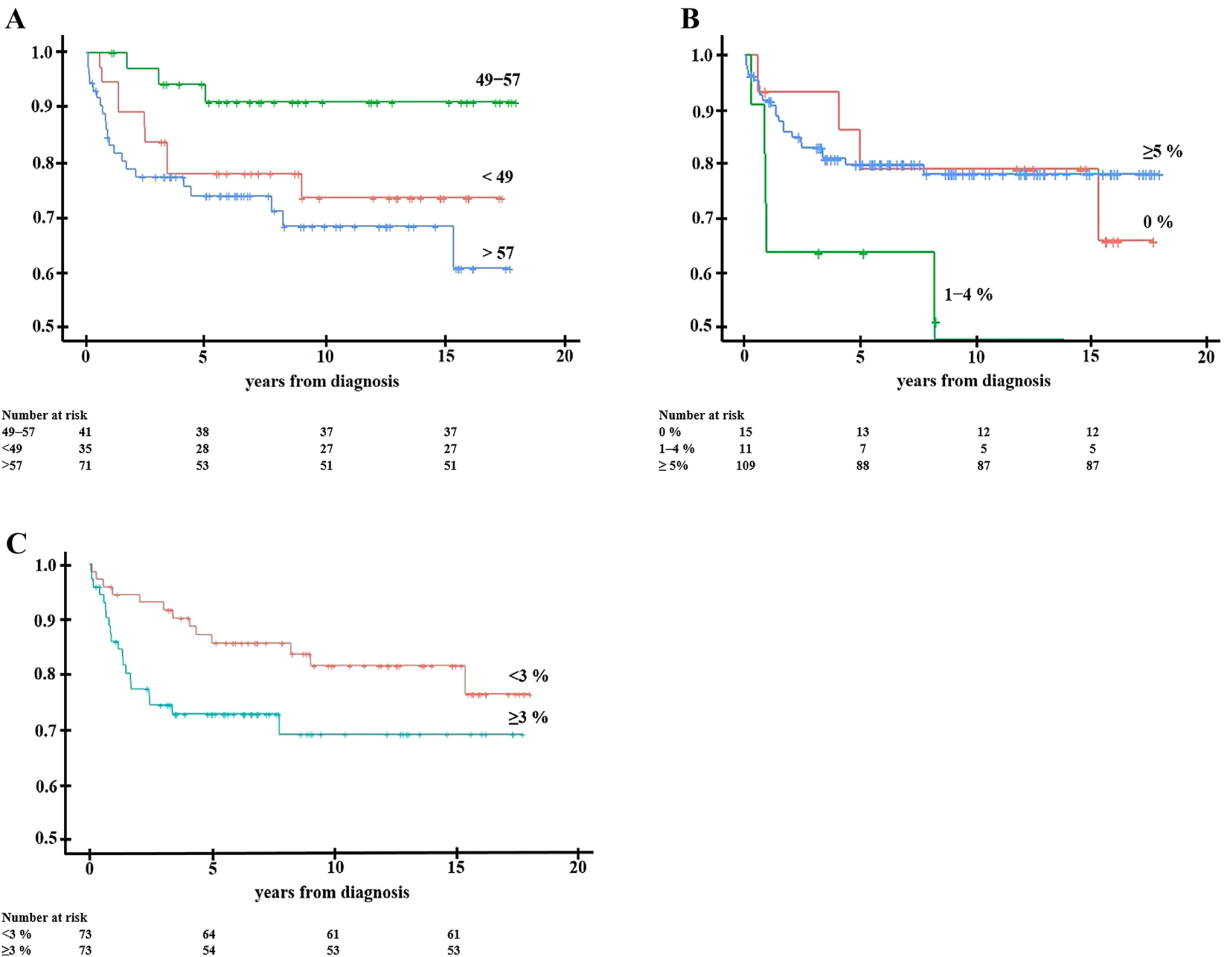
Kaplan–Meier curves presenting survival probabilities and numbers at risk for patients < 49, 49–57 and > 57 years of age at diagnosis (**A**), and tumors with high (≥ 5% of cancer cells) versus low (1–4%) vs negative geminin (**B**), and high (≥ 3% of cancer cells) versus low separase (**C**) immunoexpression

In our material of TNBC patients, age at diagnosis strongly influenced the prognostic evaluations of disease-specific mortality (Table 3). In our results, each 10-year increase in patient’s age at diagnosis indicated a 1.5-fold increased risk of breast cancer death (CI 1.2–1.8). However, age at diagnosis did not show a linear association with disease outcome. Instead, patients aged 49 − 57 showed a more favorable outcome than the younger and older age groups (Fig. 2). The risk of breast cancer death for younger patients (< 49 years) was 3.2-fold (*p* = 0.09) and for older (> 57 years) patients 4.3-fold (*p* = 0.02, CI 1.6–16.2) increased as compared to the risk of middle-aged patients. Particularly, the survival of patients > 73 years at diagnosis differed significantly from all younger age groups (HR 3.6, *p* < 0.001, CI 1.4–12.3).

**Table 3:** Prognostic impact of the studied clinical features, lack of basal differentiation and Ki-67 proliferation marker in TNBC

		$\frac{{\text{All patients}}}{{\left( {{\text{n}}\, = \,{147}} \right)}}$			$\,\frac{{ < \,{\text{49y}}}}{{\left( {{\text{n}}\, = \,{29}} \right)}}$		$\frac{{{49} - {\text{57y}}}}{{\left( {{\text{n}}\, = \,{47}} \right)}}$		$\frac{{\, > \,{\text{57y}}}}{{\left( {{\text{n}}\, = \,{71}} \right)}}$	
	HR	p	95%CI	HR	p	95%CI	p	HR	p	95%CI
Univariate										
Age	1.0	0.002	1.2–1.8		ns		ns	1.1	0.002	1.0–1.1
Nodal status		ns			ns		ns		ns	
Tumor size (continuous)	1.0	< 0.001	1.2–1.5		ns		ns	1.1	< 0.001	1.0–1.1
Tumor size (< 20 vs ≥ 20)		ns			ns		ns	3.0	0.03	1.2–5.6
Lack of basal differentiation		ns		4.4	0.03	3.2–6.2	ns		ns	
Ki-67 (continuous)		ns			ns		ns	1.0	0.009	1.0–1.1
Multivariate with age										
Nodal status	2.0	0,05	1.0–4.1		ns		ns	2.5	0.04	1.0–6.2
Tumor size (continuous)	1.0	0.0003	1.1–1.9		ns		ns	1.1	0.001	1.0–1.2
Tumor size (< 20 vs ≥ 20)	2.2	0.05	1.1–4.8		ns		ns		ns	
Lack of basal differentiation		ns		4.5	0.03	1.1–17.8	ns		ns	
Ki-67 (continuous)	1.0	0.01	1.0–1.1		ns		N/A	1.0	0.009	1.0–1.1

*ns* not significant, *N/A* not applicable

In our material, axillary lymph node status and tumor size predicted mortality in TNBC. Specifically, each 10 mm increase in tumor diameter indicated 6.1-fold increased risk of cancer death among all TNBC patients (*p* = 0.0003, CI 1.4–22.3), and 8.2-fold increased risk for patients aged > 57 at diagnosis (*p* = 0.001, CI 1.6–25.6). Concerning nodal status, axillary lymph node metastasis indicated 2.0-fold increased risk of cancer death among all TNBC patients (*p* = 0.05, CI 1.0–4.1), and 2.5-fold increased risk among patients aged > 57 at diagnosis (*p* = 0.04, CI 1.0–6.2). Among patients aged < 49 at diagnosis, only basal differentiation of the cancer cells showed statistically significant prognostic value, even after including nodal status and tumor size in the analysis (*p* = 0.04). This finding suggests that among young TNBC patients, lack of basal differentiation indicates a 4.5-fold increased risk of mortality (*p* = 0.03, CI 1.3–17.3).

Among the studied proliferation markers, Ki-67 sparsely failed to predict survival of TNBC (*p* = 0.07) but showed statistical significance after excluding the strong prognostic impact of age from the analysis (Table 3). Our findings suggest that each 10% increase in Ki-67 results in 1.6-fold increased risk of cancer death (*p* = 0.01, CI 1.2–1.7). High Ki-67 was also significantly associated with increased mortality among older patients (> 57 years of age) but not among the younger patient groups. Specifically, for patients aged > 57, each 10% increase in Ki-67 indicated 2.9-fold risk of breast cancer death (*p* = 0.009, CI 1.1–6.7). In further multivariate analyses involving age with different combinations of clinically applied prognostic features, Ki-67 (*p* = 0.02), nodal status (*p* = 0.03), and tumor size (*p* < 0.001), but not basal differentiation, predicted mortality in TNBC. In similar analyses among the subgroup of young patients, tumor size (*p* = 0.03) and basal differentiation (*p* = 0.02), but not nodal status or Ki-67, predicted disease outcome. Instead, among the oldest (> 57) patient group, tumor size (*p* = 0.03) and Ki-67 (*p* = 0.008) showed a prognostic value, but not nodal status or basal differentiation.

Among the studied novel proliferation markers, only geminin and separase showed prognostic potential in TNBC. Low geminin immunoexpression (< 5%) suggested doubled risk of breast cancer death, but the association sparsely failed statistical significance (*p* = 0.07). More specifically, the subgroup of patients exhibiting geminin immunoexpression in 1 − 4% of cancer cells showed a significantly worse outcome (HR 3.1, *p* = 0.01, CI 1.2–7.6) than the subgroup expressing geminin in ≥ 5% of cancer cells (Fig. 2). When analyzed together with patient’s age at diagnosis, the immunoexpression of geminin lost its prognostic value. Instead, among TNBC with small tumor size (< 2 cm in diameter), low geminin (< 5%) expression indicated 6.2-fold increased breast cancer mortality (p = 0.03, CI 1.4–25.3). Similarly, among node-negative patients, low geminin indicated 2.9-fold increased risk of breast cancer mortality (*p* = 0.03, CI 1.1–8.0). High separase (≥ 3% of cancer cells) suggested a 2.0-fold increased risk of breast cancer death, approaching statistical significance (*p* = 0.06) (Fig. 2). Even after excluding the impact of age at diagnosis, separase did not show significant prognostic associations. PLK1, aurora A, securin, MCM2, cyclinB1 or CDK1-3 showed no prognostic value in our material of TNBCs after testing as continuous variables or classified at several relevant cut-points.

## Discussion

**Fig. 3:**
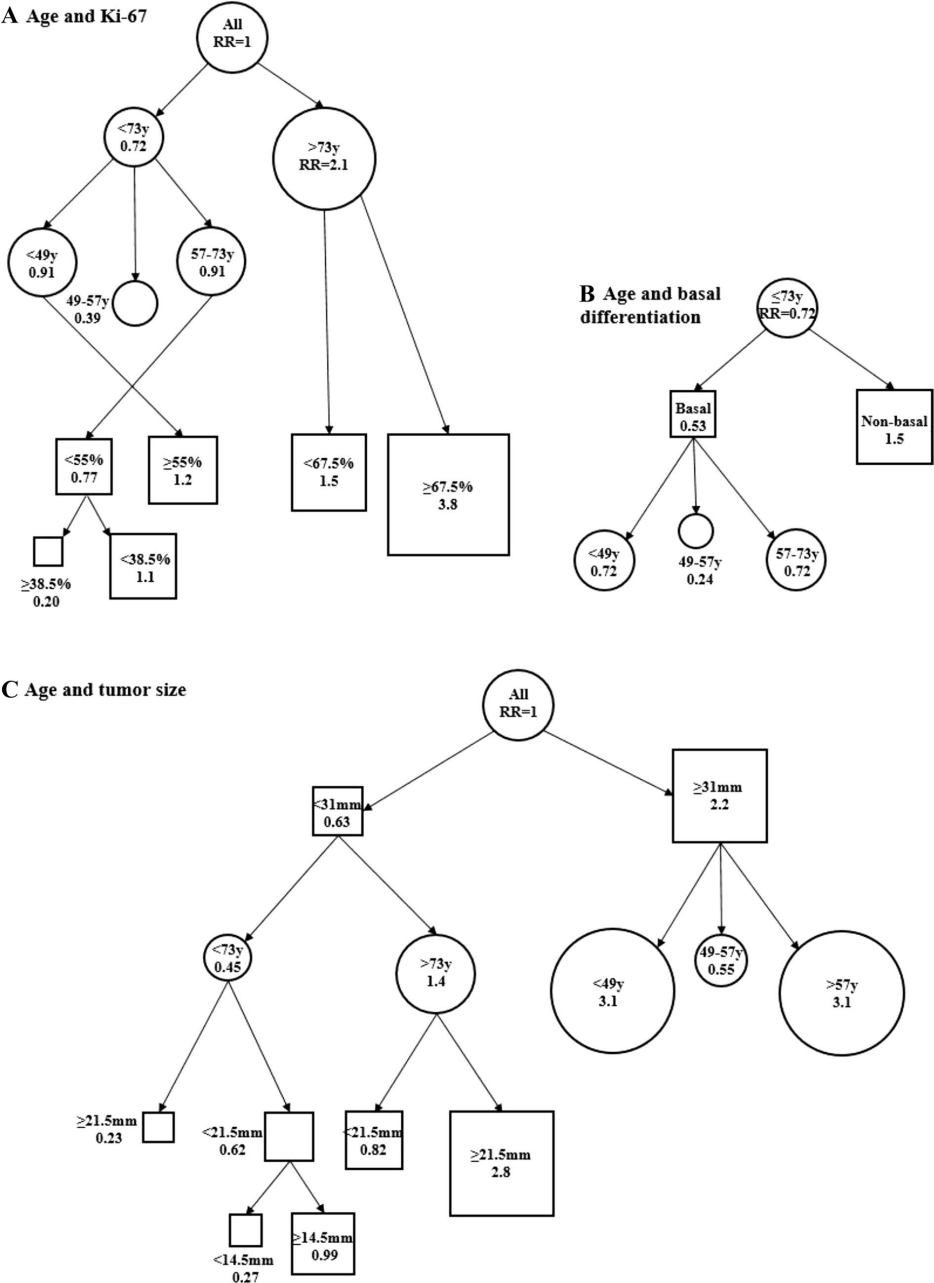
The survival trees demonstrate the relative risk (RR) of breast cancer-specific mortality in different age groups as compared to the whole material (RR = 1.0). The figures represent the proportions of mortality risk associated with age (years, y) alone (**A**, circles) and age combined with Ki-67 labeling index (**A**, squares), basal differentiation (**B**, squares) and tumor size (**C**, squares). In our results, age, Ki-67, tumor size and basal differentiation appear to have an unlinear prognostic impact in TNBC. The observation warrants a more detailed consideration of these prognostic features in personalized treatment decisions

Age at diagnosis turned out to be the decisive prognostic factor in our material of TNBCs. We observed the most favorable outcome among patients aged 49–57 years. As compared to this age group, the risk of breast cancer-specific mortality was increased threefold among the younger patients. Overrepresentation of aggressive course of disease among young TNBC patients is also reported in previous literature [26–29], especially among very young patients aged < 35 years at diagnosis [30, 31]. The prognostic role of age in TNBC has not been settled [32–38], and a considerable part of the research is controversial [39–42]. Among patients older than 57 years at diagnosis, our results indicated a fourfold increased risk of breast cancer-specific mortality, and the risk was further accentuated among the eldest patients (aged > 73) (Fig. 3A). Likewise, the majority of previous research associates increasing age with decreasing survival in TNBC [37, 43–45], with the highest reported mortality among the oldest old [46]. The survival deficit of elderly patients has been accounted for the lack of organized screening in the age group [47], as well as comorbidities [27, 48], and the consequent less intensive treatments [49]. Also, it has been suggested that the variation of outcomes in different age groups may partly be explained by the distribution of biological TNBC subtypes [37, 44, 50, 51].

Basal-like subtype is overrepresented among young TNBC patients and characterized by BRCA mutations [52–54]. However, the prognostic role of basal differentiation in TNBC is not completely settled [52, 55, 56] and part of literature reports similar prognosis for basal and non-basal TNBC [54]. Our findings (Fig. 3), however, are in line with research reporting age-dependent outcome of TNBC, and higher survival and lower recurrence rates for basal-like than non-basal-like disease [42, 50]. In our material, lack of basal differentiation was a statistically significant prognostic feature only among the youngest patient subgroup (women aged < 49), predicting more than fourfold increased risk of TNBC-specific mortality (Table 3). Apart from previous literature [50], in our material basal differentiation did not show prognostic significance among the older patients. Heterogeneity of TNBC has been presented as an explanation for the observed survival differences between age groups [3, 57, 58]. Among the young, aggressive course of disease has been explained by the lower proliferation rates in non-basal-like TNBC predisposing chemotherapy insensitivity [50], whereas among the older patients, alterations in tumor microenvironment have been addressed [32].

Regarding the established prognostic factors of breast cancer, in our material tumor size indicated up to 2.2-fold mortality risk in age-dependent multivariate analysis (Table 3, Fig. 3). Ample evidence from previous literature indicates the independent prognostic role of tumor size in TNBC [30, 59, 60], but the impact of tumor size in different age groups has not been settled. In our material, tumor size was a significant predictor of survival only among the subgroup of patients > 57, but not > 73 years at diagnosis. This finding may be explained by the Finnish national breast cancer screening program [61] which has resulted in a significant improvement of breast cancer survival, particularly among TNBC patients [62]. Among the elderly, the more sinister outcome may also reflect the complexity of chemotherapy decision-making due to the heterogeneity of patient characteristics, presence of comorbidities and increased risk of toxicity [63].

According to general understanding, TNBC has a reduced probability of lymph node involvement, due to its tendency to spread hematogenously [64]. In our material, nodal status alone did not predict disease survival (Table 3). However, after standardizing for age at diagnosis, axillary metastasis proved to be an independent prognostic factor in the whole material and among patients aged > 57 years, indicating 2.0- and 2.5-fold increased mortality, respectively. Among younger patients, instead, age was such a strong prognostic factor that it overrode the impact of nodal status. In previous literature, no consensus reigns on the prognostic impact of nodal status in TNBC. Part of previous research considers axillary metastases as an adverse clinical feature [65, 66], especially for short-term survival [67]. The association between nodal status and outcome of TNBC, however, appears not to be straightforward [67, 68], while even extensive metastatic disease has not been observed to impair survival [69]. The discrepant and confusing association between nodal status and disease outcome have been linked to the genetic and clinical heterogeneity of TNBC [67].

Defects in cell cycle regulation and DNA repair mechanisms are common characteristics of TNBC, and enhanced cell cycle is recognized as a specific cellular feature of young patients [65, 70]. This is also reflected in proliferation indices, such as immunohistochemically detected Ki-67 labeling index, which has been reported to decrease with increasing age [70]. Similar to our present findings, the majority of researchers have associated high Ki-67 with unfavorable disease outcome among TNBC patients [9, 13, 60, 71]. In our material, however, Ki-67 was an independent prognosticator only among patients aged > 57 years for whom each 10% increase in Ki-67 indicated 2.9-fold increased risk of mortality. As previously reported [72], Ki-67 was not a clinically applicable prognostic factor among the youngest patient subgroup in our material, possibly reflecting the age-dependent distribution of different genetic and transcriptional phenotypes in TNBC [27, 32, 70]. However, the clinical applicability of Ki-67 in predicting the outcome of TNBC is impaired by different areas assessed, such as hot spot or average across slide [73], and the varying criteria for high vs low labeling index with cut-points ranging from 14% [1] to 25%, and even up to 60% [11, 72, 74]. Moreover, poor understanding of the functions and dynamics of Ki-67 protein during the cell cycle hamper detailed prognostic conclusions [75].

Previous literature has identified various cell cycle-regulating proteins with expression peaks at specific steps of cell division. Due to their distinct expression patterns in cell cycle, these proteins have been suggested with advantages over Ki-67 in achieving more detailed prognostic information [6]. Previously, based on invasive breast carcinomas of all intrinsic subtypes, we have reported 8.4-fold increased risk of mortality associated with the combination of separase, securin and cdk1, each a crucial regulator of cell cycle-progression [76]. Contradictory to our previous results, apart from geminin, all the tested novel biomarkers for cell proliferation failed to show statistically significant prognostic associations in our present material of TNBCs.

Geminin, a DNA replication inhibitor, is activated during cell cycle-progression by the anaphase-promotion complex (APC) leading to initiation of sister chromatid separation [77]. The specific role of geminin is to limit DNA replication and trigger sister chromatid separation and, therefore, it is considered to qualify exceptionally well as a cell cycle-specific proliferation marker [78–80]. In previous literature, loss of geminin expression has been observed to predict poor survival in breast cancer [81–83] and aggressive course of disease in the subgroup of TNBCs [84–86]. In our material, geminin identified TNBC patients with increased mortality among subgroups generally associated with favorable outcome, i.e. patients with node-negative disease and small tumor size. Consistent with previous literature, adverse outcome in TNBC was associated with geminin expression but the association was not linear. Emerging evidence emphasizes the multifaceted role of geminin in malignancy [78, 87]. Based on the current understanding, geminin overexpression arrests cell proliferation whereas loss of geminin may generate re-replication, predisposing cancer cells to aneuploidy and drug-resistance [81, 88].

In summary, our results emphasize the nature of TNBC as a heterogeneous disease with varying courses of disease in different age groups. Traditional clinical features do not provide optimal prognostic characterization for all TNBC patients, and young age should be considered as an additional adverse prognostic feature. Contrary to other breast cancer subtypes, proliferation does not explain for the survival differences in all age-subgroups of TNBC patients. Among the youngest patients (< 49 years at diagnosis), lack of basal differentiation predicted disease outcome. The traditional prognostic features of breast cancer, tumor size and nodal status, applied only among the oldest patient group (> 57 years at diagnosis). However, among middle-aged patients (49–57 years at diagnosis), no features indicating disease outcome could be detected in our material. The findings provide additional support for the hypothesis that young TNBC patients comprise a unique disease entity, while elderly patients represent a more coherent subgroup which, in this therapeutically challenging disease, may indicate potential regarding future targeted treatments. While the explanation for the survival deficit between different age groups in TNBC still remains unsettled, it is evident that TNBC diagnosed at a young age warrants individual therapeutic considerations, especially concerning the increasing number of patients receiving neoadjuvant treatment.

## Data Availability

The datasets generated during and/or analyzed during the current study are not publicly available due to the patients’ privacy/ethical restrictions but are available from Auria Biobank, Turku University Hospital, Turku, Finland (www.auria.fi) on reasonable request.
